# Prediction of hepatic necroinflammatory activity in patients with chronic hepatitis B by a simple noninvasive model

**DOI:** 10.1186/s12967-018-1538-z

**Published:** 2018-06-18

**Authors:** Fei-Fei Shen, Yan Wang, Yi-Fei Wang, Rui-Dan Zheng, Jian-Chun Xian, Jun-Ping Shi, Ying Qu, Yu-Wei Dong, Ming-Yi Xu, Lun-Gen Lu

**Affiliations:** 1 Department of Gastroenterology, Shanghai General Hospital, Shanghai Jiao Tong University School of Medicine, Shanghai, China; 2 Research and Therapy Center for Liver Diseases, Zhengxing Hospital, Zhangzhou, Fujian Province China; 3 Department of Infectious Disease, Taizhou People’s Hospital, Taizhou, Jiangsu Province China; 4 Research and Therapy Center for Liver Diseases, Hangzhou Second People’s Hospital, Hangzhou, Zhejiang Province China

**Keywords:** Chronic hepatitis B, Hepatic necroinflammatory activity, Noninvasive, Prediction

## Abstract

**Background:**

A model was constructed using clinical and serum variables to discriminate between chronic hepatitis B (CHB) patients with and without significant necroinflammatory activity (score 4–18 vs. score 0–3).

**Methods:**

Consecutive CHB patients who underwent liver biopsy were divided into two sequential groups: a training group (n = 401) and a validation group (n = 401). Multivariate analysis identified alanine aminotransferase, γ-glutamyltransferase, prothrombin time and albumin as independent predictors of necroinflammatory activity.

**Results:**

The area under the receiver operating characteristic curve was 0.826 for the training group and 0.847 for the validation group. Using a cut-off score of H ≤ 0.375, significant necroinflammatory activity (score 4–18) was excluded with high accuracy [78.2% negative predictive value (NPV), 72% positive predictive value (PPV), and 90.8% sensitivity] in 238 (59.4%) of 401 patients in the training group and with the same certainty (88.1% NPV, 61.2% PPV, and 95.1% sensitivity) among 204 (50.9%) of 401 patients in the validation group. Similarly, applying a cut-off score of H > 0.720, significant necroinflammatory activity was correctly identified with high accuracy (90.8% PPV, 57.7% NPV, and 92.0% specificity) in 150 (37.4%) of 401 patients in the training group and with the same certainty (91.8% PPV, 64.6% NPV, and 95.4% specificity) in 188 (46.9%) of 401 patients in the validation group.

**Conclusions:**

A predictive model based on easily accessible variables identified CHB patients with and without significant necroinflammatory activity with a high degree of accuracy. This model may decrease the need for liver biopsy for necroinflammatory activity grading in 72.1% of CHB patients.

## Background

Hepatitis B virus (HBV) infection remains a worldwide public health problem with high morbidity and mortality. Approximately 2 billion people have been infected with HBV, and approximately 240 million people are chronic hepatitis B surface antigen (HBsAg) carriers [1, 2]. Approximately 7.2% of people in China have been infected with HBV [3]. The number of HBV-related deaths caused by liver failure, liver cirrhosis and/or hepatocellular carcinoma increased by 33% between 1990 and 2003. In 2013, there were > 686,000 HBV-related deaths worldwide [4]. The incidence of HBV-related liver cirrhosis and HCC is 30 and 60% worldwide and 45 and 80% in China, respectively [5, 6]. The annual rate of decompensated cirrhosis is approximately 3–5%, and the 5-year cumulative incidence is approximately 16% in patients with chronic hepatitis B (CHB) [7]. The 5-year mortality rate of CHB with compensated and decompensated cirrhosis is 14–20% and 70–86%, respectively.

For many years, liver biopsy has been the gold standard for diagnosing steatosis, fibrosis, and necroinflammatory activity in liver tissues. Because of its invasive nature, both patients and doctors may prefer to avoid a liver biopsy procedure. In addition, the procedure has some disadvantages, for example, intra- and interobserver inconsistencies may arise, dynamic observation and follow-up are difficult, and the size of the specimens obtained is sometimes inadequate. For these reasons, the gold standard of liver biopsy can result in misdiagnosis of cirrhosis in 10–30% of patients [8]. Therefore, a convenient and reliable noninvasive diagnostic index or method for evaluating necroinflammatory activity and fibrosis is needed to replace liver biopsy. Optimal methods for the diagnosis of liver disease should be noninvasive, low cost, and easy to reproduce and should have a high sensitivity, specificity and accuracy.

Several diagnostic models, such as the classic aminotransferase to platelet ratio index (APRI) (aspartate aminotransferase [AST]  × 100/platelet count) [9], the Forns index [10], the Fibrotest [11], the Shanghai liver fibrosis group (SLFG) model [12], Ho’s model (α2-macroglobulin, vitamin D binding protein, and apolipoprotein A1) [13] and the S index [γ-glutamyltransferase (GGT), platelet count, and albumin (ALB)] [14], have been used to diagnose liver fibrosis with high accuracy, sensitivity and specificity. However, few noninvasive diagnostic models have been established for the study of chronic hepatitis necroinflammatory activity. The ActiTest diagnosis model was established for chronic hepatitis C (CHC) [15]. Although CHB and CHC are both of viral origin, there are significant differences between the two in etiology, natural history, histopathology and treatment. We assessed the medical histories, physical examinations and blood test results in CHB patients and constructed and validated a model and scoring system by combining the Knodell histologic activity index (HAI) score [16] with routine laboratory tests to distinguish patients with and without significant necroinflammatory activity. This model may render liver biopsy unnecessary in a considerable proportion of CHB patients.

## Methods

### Patients

Between July 2006 and December 2012, 978 consecutive patients with HBV infections from five hospitals (Shanghai First People’s Hospital, Shanghai Jiaotong University School of Medicine; Zhengxing Hospital, Zhangzhou, Fujian Province; Taizhou People’s Hospital, Jiangsu Province; Hangzhou Second People’s Hospital, Zhejiang Province, and Zhoushan People’s Hospital, Zhejiang Province) in China were recruited for this study. The inclusion criteria were CHB patients aged 18–65 years who were positive for serum HBsAg and/or HBV DNA for 6 months or more before enrollment. The exclusion criteria were decompensated cirrhosis; coinfection with HIV or hepatitis C virus (HCV); having received antiviral treatment; taking immunoregulation drugs such as cytotoxic agents and hormones; using drugs, such as traditional Chinese medicines, capable of reducing serum liver enzyme activity and bilirubin levels; alcohol consumption > 30 g/day; autoimmune disease or antinuclear antibody titers higher than 1:160; and other chronic liver disease. The study was approved by the Ethics Committee of Shanghai First People’s Hospital, Shanghai Jiao Tong University School of Medicine. Informed consent to participate in the study was obtained from each patient.

### Serum markers

Blood samples were obtained from all patients on the day before liver biopsy. Serum markers were measured in either fresh blood or frozen serum samples stored at − 40 °C. Hematological (Sysmex XE-2100, Sysmex Corporation, Japan) or common biochemical (Hitachi 7600-020 Analyzer, Hitachi, Japan; Wako Diagnostics reagents, Wako Pure Chemical Industries Ltd, Japan) and coagulation function (MC-2000 blood coagulation analyzer, Meichuang Company, Germany) tests were performed using standard methodologies. The reference value was 5–50 IU/L for alanine aminotransferase (ALT) (IFCC, 37 °C), 15–60 IU/L for GGT, 40–55 g/L for ALB, and 9–13 s for prothrombin time (PT). Hepatitis virus markers (Abbott ARCHITECT i2000 SR system, Abbott Laboratories, Abbott Park, IL, USA) including HBsAg, hepatitis B surface antibody (HBsAb), hepatitis B early antigen (HBeAg), hepatitis B early antibody (HBeAb), hepatitis B core antibody (HBcAb), and anti-HCV were measured with Clinical Laboratory Improvement Amendment (CLIA) systems. HBV DNA concentrations were measured using the COBAS TaqMan assay (Roche Molecular Systems, Branchburg, NJ, USA), which has a lower limit of quantification of 100 copies/mL. All markers described above were measured by the Department of Laboratory Medicine, Shanghai First People’s Hospital, Shanghai Jiaotong University School of Medicine, Shanghai, China.

### Liver biopsy

All patients underwent liver biopsy directed by ultrasonography within 1 week after inclusion in the study. The biopsy specimens were fixed with 10% formalin and routinely embedded in paraffin, and the tissue sections were processed with hematoxylin and eosin (HE), Masson’s trichrome, and reticular fiber staining. The liver biopsy specimens were required to be at least 1.5 cm in length and contain at least six portal tracts for diagnosis. Liver necroinflammatory activity (G0–G4) was estimated according to Scheuer’s classification. Liver necroinflammatory activity was considered significant for Knodell HAI scores of 4–18 (score 4–8: mild; score 9–12: moderate; and score 13–18: severe), while a score of 0–3 indicated no significant necroinflammatory activity [2]. Liver necroinflammatory activity was staged on a scale of 0–4: G0–1 (HAI 0–3) = nonspecific reactive hepatitis, CLH, or CPH; G2 (HAI 4–8) = severe CLH, CPH or mild CAH; G3 (HAI 9–12) = moderate CAH; G4 (HAI 13–18) = severe CAH with bridging necrosis. All sections were blindly and independently assessed by three pathologists, and the observed results were processed by the Kappa concordance test. The inter- and intraobserver agreements were excellent (P < 0.01). When the three pathologists did not agree, the specimens were re-examined to analyze discrepancies, and a consensus was reached.

### Statistical analysis

Statistical analysis was performed using SPSS V20.0 (SAS Institute Inc., Cary, NC, USA) software. The patient characteristics are expressed as the median (25th–75th percentile), and categorical data are expressed as a number (percentage). Univariate analysis (Student’s *t* test, Mann–Whitney U test or χ^2^ test) was carried out to identify variables that were significantly different between patients with and without significant necroinflammatory activity. Categorical variables were analyzed using the χ^2^ test, while continuous variables were assessed with an independent samples *t* test or the Mann–Whitney U test as appropriate. Correlation was evaluated by Spearman’s rank correlation coefficient. The predictive variables were selected by a stepwise forward analysis [likelihood ratio (LR), enter P < 0.05, remove P > 0.10] from the significant variable from the univariate analysis (P < 0.05). The model was constructed using the results of the multivariate logistic regression analysis. The diagnostic value of the model was assessed by calculating the area under the receiver operating characteristic curve (AUROC). The diagnostic accuracy was calculated using sensitivity (SEN, specificity (SPE), positive predictive value (PPV), negative predictive value (NPV), and LR.

## Result

### Patient characteristics

**Fig. 1:**
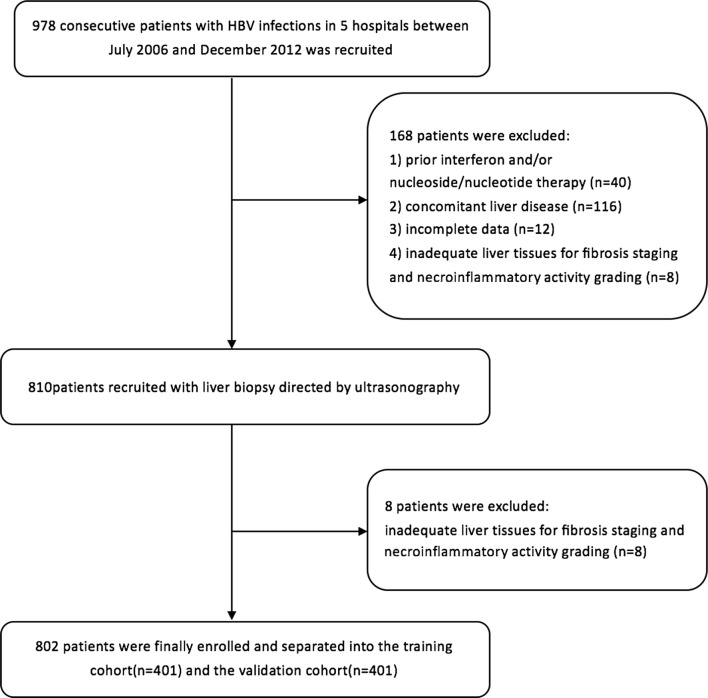
Study flow diagram of patient groups

As described above, a total of 978 patients were recruited for this study. According to the inclusion and exclusion criteria, 176 patients were excluded because of prior interferon and/or nucleoside/nucleotide therapy, concomitant liver disease, inadequate liver tissues for fibrosis staging and necroinflammatory activity grading, or incomplete data. Eventually, a total of 802 patients were enrolled in the study and separated into two groups, the training cohort and the validation cohort (Fig. 1). Consecutive patients who were biopsied between July 2006 and October 2009 comprised the training group. Patients who were biopsied between November 2009 and December 2012 comprised the validation group. The characteristics of the patients at the time of liver biopsy are shown in Table 1. There were no significant differences (P > 0.05) between the training group and the validation group in any of the variables. There was also no significant difference between the training cohort and the validation cohort in the degree of hepatic necroinflammatory activity.

**Table 1:** Baseline characteristics of the 802 patients with chronic hepatitis B at the time of liver biopsy: comparison between the training set and the validation set

Variables	The training cohort (n = 401)	The validation cohort (n = 401)	All patients (n = 802)
Age (years)	33 (26.5–41)	36 (29–42)	34 (27–41)
Sex (male n, %)	322 (80.2%)	318 (79.3%)	640 (79.8%)
Weight (kg)	62 (56–68)	61 (56–66)	61 (56–67)
RBC (10^12^/L)	4.8 (4.3–5.5)	4.6 (4.3–5)	4.7 (4.3–5.3)
WBC (10^9^/L)	5.13 ± 1.23	5.24 ± 1.41	5.19 ± 1.32
PLT (10^9^/L)	144 (110–193)	138 (111–176)	140 (110–86.25)
HB (g/L)	140 (130–153)	139 (125–150.5)	139 (128–152)
TG (mmol/L)	1.2 (0.9–1.5)	1.2 (0.9–1.4)	1.2 (0.9–1.4)
TC (mmol/L)	4.2 (3.6–4.9)	4.2 (3.6–4.65)	4.2 (3.6–4.7)
ALT (IU/L)	75 (50–142.5)	62 (42–101.5)	68 (45–121)
AST (IU/L)	56 (37.5–101.5)	50 (34–75)	51 (35–86)
GGT (IU/L)	54 (29–111)	56 (35–82)	54 (32–93)
ALP (IU/L)	79 (64.5–108)	79 (64–100)	78 (63.75–104.25)
TB (μmol/L)	14.5 (10.95–20.2)	15.3 (12.3–19.85)	15 (11.5–20.125)
DB (μmol/L)	5 (3.5–8.3)	5 (3.9–6.95)	5 (3.6–7.4)
ALB (g/L)	41 (37.85–46)	41 (38–44)	42 (38–45.1)
AFP (ng/mL)	3.8 (2.6–7.25)	4.2 (3.05–5.9)	3.9 (2.8–6.7)
PT (s)	12.5 (11.75–13.4)	12.3 (11.7–13.2)	12.3 (11.7–13.2)
HBVDNA (log_10_ copies/mL)	5.69 (3.34–6.92)	6.37 (3.67–7.52)	5.84 (3.41–7.08)
Grading of liver necroinflammatory activity			
G0–1:HAI (0–3) (n, %)	163 (40.6%)	170 (42.4%)	333 (41.5%)
G2:HAI (4–8) (n, %)	119 (29.7%)	116 (28.9%)	235 (29.3%)
G3:HAI (9–12) (n, %)	91 (22.7%)	88 (21.9%)	179 (22.3%)
G4:HAI (13–18) (n, %)	28 (7.0%)	27 (6.7%)	55 (6.9%)

The patient characteristics are expressed as the median (25th–75th percentile), and categorical data are expressed as a number (percentage). There were no significant differences between the training group and the validation group in any of the variable

*RBC* red blood cell, *WBC* white blood cell, *PLT* platelets, *HB* haemoglobin, *TG* triglyceride, *TC* total cholesterol, *ALT* alanine aminotransferase, *AST* aspartate aminotransferase, *GGT* γ-glutamyl transpeptidase, *ALP* alkaline phosphatase, *TB* total bilirubin, *DB* indirect bilirubin, *ALB* albumin, *AFP* α-fetal protein, *PT* prothrombin time

### Predictors of necroinflammatory activity

The training cohort was divided into patients without significant necroinflammatory activity (scores of 0–3) and patients with significant necroinflammatory activity (scores of 4–18) based on HAI scores. Variables that were determined to be associated with the presence of significant necroinflammatory activity (scores of 4–18) in the training cohort (401 patients) by univariate analysis are shown in Table 2.

**Table 2:** Variables associated with the presence of significant necroinflammatory activity (score 4–18) in the training group (401 patients) by univariate analyses cohort

Variables	No significant inflammation (n = 163)	Significant inflammation (n = 238)	P value
Age (year)	32 (25–40)	34 (27–41.25)	0.114
Weight (kg)	64 (58–69)	61 (56–66)	0.013
Male (n)	128 (78.5%)	194 (81.5%)	0.523^b^
RBC (10^12^/L)	4.8 (4.2–5.4)	4.8 (4.3–5.5)	0.119
WBC (10^9^/L)	5 (4.5–5.625)	4.95 (4.3–5.5)	0.056
PLT (10^9^/L)	147 (110–193)	141.5 (109–192)	0.78
HB (g/L)	141 (131–154)	139.5 (129–152.25)	0.533
TG (mmol/L)	1.2 (0.8–1.5)	1.2 (0.9–1.5)	0.793
TC (mmol/L)	4.3 (3.9–5)	4.1 (3.5–4.7)	0.005
ALT (IU/L)	64 (42–85)	95.5 (55–209.75)	< 0.001^a^
AST (IU/L)	46 (30–67)	72 (42–139)	< 0.001^a^
GGT (IU/L)	32 (20–54)	108.57 ± 94.57	< 0.001^a^
ALP (IU/L)	72 (60–88)	87.5 (70–117)	< 0.001^a^
TB (μmol/L)	12.9 (10.4–17.6)	15.9 (11.4–24.45)	< 0.001^a^
DB (μmol/L)	4.4 (3.2–6)	5.85 (3.9–9.7)	< 0.001^a^
ALB (g/L)	45 (41–48.2)	39 (36–43.725)	< 0.001
AFP (ng/mL)	3 (2.3–4.5)	4.75 (3.1–11)	< 0.001^a^
PT (s)	12.1 (11.5–12.8)	12.8 (12–13.725)	< 0.001^a^
HBVDNA (log_10_ copies/mL)	5.43 (3–6.64)	5.8 (4.08–6.98)	0.063^a^

*RBC* red blood cell, *WBC* white blood cell, *PLT* platelets, *HB* haemoglobin, *TG* triglyceride, *TC* total cholesterol, *ALT* alanine aminotransferase, *AST* aspartate aminotransferase, *GGT* γ-glutamyl transpeptidase, *ALP* alkaline phosphatase, *TB* total bilirubin, *DB* indirect bilirubin, *ALB* albumin, *AFP* α-fetal protein, *PT* prothrombin time

^a^ Values are comparisons between the training and validation set using an independent samples t test, except nonparametric test

^b^ Values are comparisons between the training and validation set using an independent samples t test, except x^2^ test

Variables including weight, TC, ALT, AST, GGT, ALP, TB, DB, ALB, AFP and PT were identified as predictors of necroinflammatory activity by univariate analysis (Table 2).

### Establishment and evaluation of the predictive model

The indexes associated with significant necroinflammatory activity in the training group were screened to establish the predictive model, which was constructed by stepwise forward logistic regression analysis (Table 3). The formula acquired was G = Exp (1.214 + 0.006 × ALT (IU/L) + 0.008 × GGT (IU/L) + 0.324 × PT (s) − 0.143 × ALB (g/L)). The formula was converted as follows: H index = G/(G + 1).

**Table 3:** Predicotors of significant necroinflammatory activity according to stepwise forward logistic regression analysis

Parameter	B	S.E	Wals	df	Sig.	Exp (B) (95% CI)
ALT	0.006	0.002	9.994	1	0.002	1.004 (1.002, 1.009)
GGT	0.008	0.002	11.859	1	0.001	1.008 (1.004, 1.013)
ALB	− 0.143	0.024	36.274	1	0.000	0.867 (0.827, 0.908)
PT	0.324	0.108	8.988	1	0.003	1.383 (1.119, 1.710)
Constant	1.214	1.660	0.535	1	0.465	3.367

*ALT* alanine aminotransferase, *GGT* γ-glutamyl transpeptidase, *ALB* albumin, *PT* prothrombin time

**Fig. 2:**
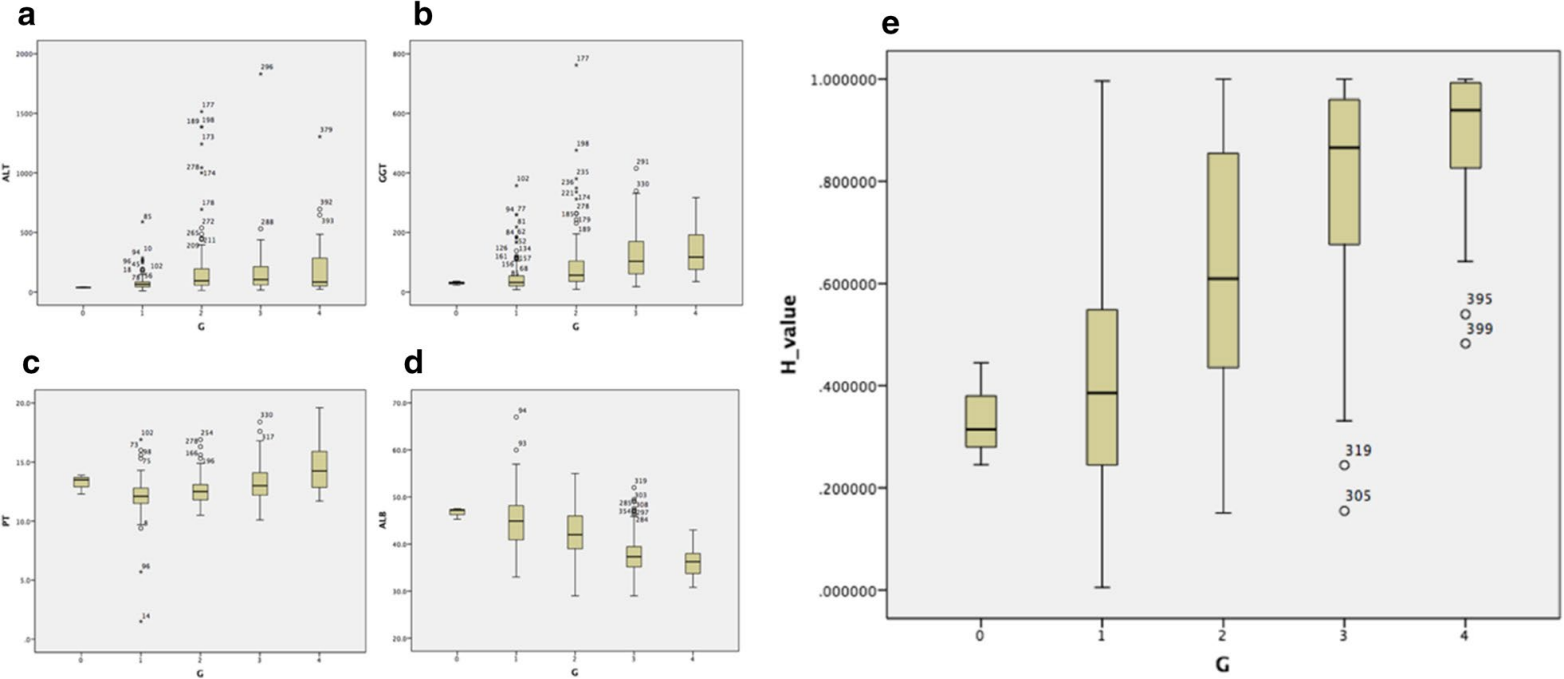
The correlation of values and hepatic necroinflammatory activity with biopsy. The correlations of values (**a** ALT, **b** GGT, **c** PT, **d** ALB, **e** H value) and hepatic necroinflammatory activity with biopsy are shown in box plots. The top and bottom of each box represents the 25th and 75th centile intervals. The line through the box in the median

Correlations of hepatic necroinflammatory activity with biopsy and the H index calculated by the model we constructed were evaluated by Spearman’s rank correlation coefficient. The correlation coefficient r was significant (r = 0.625, P < 0.01), whereas the correlation coefficients of hepatic necroinflammatory activity with biopsy and serum biomarkers (ALT, GGT, PT and ALB) were 0.291, 0.525, 0.360 and − 0.525, respectively. The correlations of the abovementioned values (H index, ALT, GGT, PT and ALB) and hepatic necroinflammatory activity with biopsy are shown in box plots (Fig. 2).

**Fig. 3:**
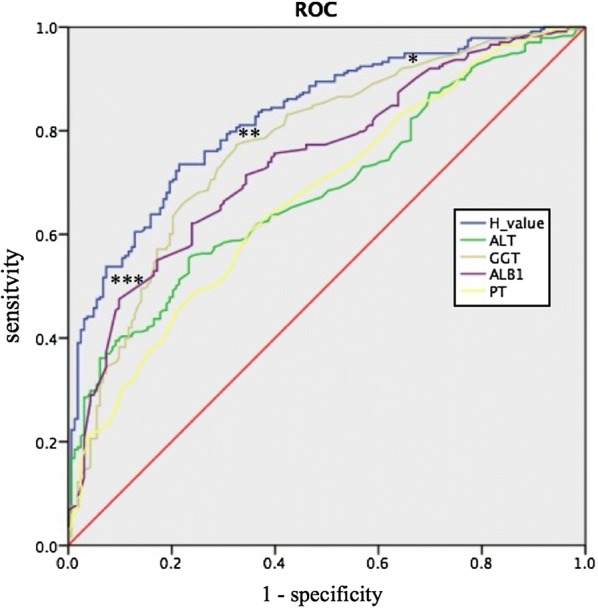
ROC curves of the model in the training cohort. The AUROC of the H index, ALT, GGT, ALB, and PT was 0.826 (95% CI 0.786–0.866, P < 0.001), 0.685 (95% CI 0.634–0.736), 0.771 (95% CI 0.725–0.818), 0.744 (95% CI 0.696–0.792) and 0.672 (95% CI 0.619–0.725), respectively. *Sensitivity is 90.8%, and specificity is 48.5%, with an H index cutoff of 0.375 ***Sensitivity is 53.8%, and specificity is 92.0%, with an H index cutoff of 0.720. **Sensitivity is 83.3%, and specificity is 67.0%, with an intermediate H index cutoff of 0.560 (the max Youden index point)

The AUROC of the H index, ALT, GGT, ALB, and PT was 0.826 (95% CI 0.786–0.866, P < 0.001), 0.685 (95% CI 0.634–0.736), 0.771 (95% CI 0.725–0.818), 0.744 (95% CI 0.696–0.792) and 0.672 (95% CI 0.619–0.725), respectively (Fig. 3). The AUROC of the H index was higher than that of any single variable.

### Cut-off H index values

We selected low (0.375) and high (0.720) cut-off values according to the Youden index (Youden index = sensitivity + specificity − 1) to identify the absence and presence of significant necroinflammatory activity (Fig. 3). The diagnostic accuracy was analyzed. The SEN, SPE, PPV, NPV, positive LR (LR+) and negative LR (LR−) are shown in Table 4. In this training cohort, at an H index cut-off of 0.375, 216 of 300 (72%) patients without significant necroinflammatory activity were identified correctly. Patients with significant necroinflammatory activity could be diagnosed with a high NPV of 78%, as 79 of 101 (78.2%) patients were correctly ruled out. Therefore, more than half of the patients could be excluded from undergoing liver biopsy. At an H index cut-off of 0.720, 150 of 260 (57.7%) patients with necroinflammatory activity in the liver biopsy were correctly identified. Patients without significant necroinflammatory activity could be diagnosed with a high PPV of 91%.

**Table 4:** Diagnostic accuracy of the H value in the training and validation cohorts

	Cutoff	G0–1	≥ G2	SEN	SPE	PPV	NPV	LR+	LR−	Population involved (%)	Interpretation
Training cohort (n = 401)	Low cutoff			0.908	0.485	0.720	0.782	1.761	0.191	59.4	Absence of necroinflammatory activity (78% certainty)
	< 0.375	216	22								
	> 0.375	84	79								
	High cutoff			0.538	0.920	0.908	0.577	6.743	0.502	40.6	Presence of necroinflammatory activity (91% certainty)
	< 0.720	128	110								
	> 0.720	13	150								
Validation cohort (n = 401)	Low cutoff			0.951	0.376	0.612	*0.881*	1.523	0.130	50.9	Absence of necroinflammatory activity (88% certainty)
	< 0.375	194	10								
	> 0.375	123	74								
	High cutoff			0.495	0.954	*0.918*	0.646	10.83	0.529	49.1	Presence of necroinflammatory activity (92% certainty)
	< 0.720	101	103								
	> 0.720	9	188								

In the validation cohort, using a cut-off score of H ≤ 0.375, significant necroinflammatory activity was excluded with high accuracy (88.1% NPV and 95.1% sensitivity). Similarly, applying a cut-off score of H > 0.720, significant necroinflammatory activity was correctly identified with high accuracy (91.8% PPV and 95.4% specificity)

Italic values indicate significant positive values in the validation cohort

*SEN* sensitivity, *SPE* specificity, *PPV* positive predictive value, *NPV* negative predictive value, *LR+* positive likelihood ratio, *LR−* negative likelihood ratio

### Assessment of the predictive model in the validation cohort

**Fig. 4:**
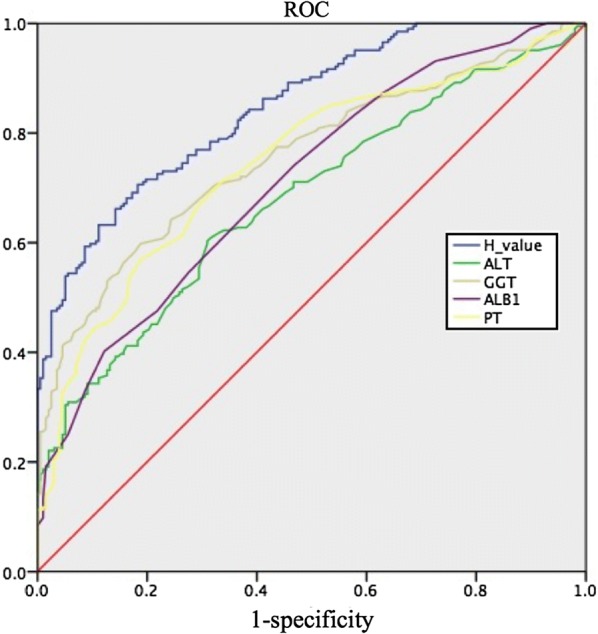
ROC curves of the model in the validation cohort. The AUROC of the H index, ALT, GGT, ALB, and PT was 0.847 (95% CI 0.811–0.883, P < 0.001), 0.679 (95% CI 0.627–0.731), 0.757 (95% CI 0.709–0.804), 0.711 (95% CI 0.662–0.761) and 0.744 (95% CI 0.696–0.792), respectively

In predicting significant necroinflammatory activity in the validation cohort, the AUROC was 0.847 for the H index (Fig. 4) and was higher than that of any single variable. Using the cut-off values of 0.375 and 0.720, the presence of significant necroinflammatory activity was predicted with high sensitivity (95%) and high specificity (95.4%) in the validation cohort (Table 4). In the validation cohort, at an H index cut-off of 0.375, 194 of 317 (61.2%) patients without significant necroinflammatory activity were identified correctly. Patients with significant necroinflammatory activity could be diagnosed with a high NPV of 88%, as 74 of 84 (88.1%) patients were correctly ruled out. Therefore, almost half of the patients could be excluded from undergoing liver biopsy. At an H index cut-off of 0.720, 188 of 291 (64.6%) patients with necroinflammatory activity in the liver biopsy were correctly identified. Patients without significant necroinflammatory activity could be diagnosed with a high PPV of 92%. Only 9 of 110 (8.1%) patients without significant necroinflammatory activity were classified incorrectly.

## Discussion

Globally, an estimated 240 million individuals have CHB; the prevalence varies geographically and is highest in Africa and Asia [2]. In China, a national survey of HBV seroepidemiology has already shown a decrease in the general prevalence of HBsAg, from 9.75% in 1992 to 7.18% in 2006, and a decrease in children less than 5 years of age, from 9.67% in 1992 to 0.96% in 2006 [5]. Death from cirrhosis and hepatocellular carcinoma (HCC) has been estimated at 310,000 and 340,000 per year, respectively [16]. The goal of HBV therapy is to prevent liver-related morbidity and mortality. Patients in the immune-active phases of infection (HBeAg-positive or HBeAg-negative) display elevated ALT, histological evidence of liver injury (significant necroinflammatory activity and/or fibrosis), and elevated HBV DNA levels, with a greater risk of progressive liver disease and its associated complications [17]. Significant necroinflammatory activity and liver tissue fibrosis are risk factors for these complications and are indications for therapeutic intervention.

The American Association for the Study of Liver Diseases (AASLD) [16], European Association for the Study of the Liver (EASL) [18], and Asian Pacific Association for the Study of the Liver (APASL) [19, 20] guidelines for treatment of CHB patients specify that the presence of moderate to severe necroinflammatory activity in liver biopsy specimens is an indication for antiviral therapy. At present, no single noninvasive indicator can be used to accurately diagnose and assess pathological changes in CHB patients. Due to the severity of liver disease, the degree of fibrosis and necroinflammatory activity should be determined to ensure that patients receive effective antiviral treatment.

In recent years, many studies have aimed to identify an optimal model for the noninvasive diagnosis of liver fibrosis, and many combined indexes for diagnosis have been developed. However, the noninvasive assessment of liver necroinflammatory activity in CHB patients is less well studied. One representative model is the ActiTest diagnostic model, which includes alpha 2-macroglobulin, apolipoprotein A1, haptoglobin, GGT, and ALT [15]. However, the ActiTest model was established using data from CHC patients. Whether ActiTest is useful for Chinese CHB patients requires further study. Hence, it is necessary to establish a simple, low-cost model for the noninvasive diagnosis of liver necroinflammatory activity in CHB patients.

A simple diagnostic model distinguishing CHB patients with significant necroinflammatory activity from those without significant necroinflammatory activity was established in this study based on commonly used, routine clinical tests. We developed the index using factors that were independently associated with liver necroinflammatory activity obtained by routine blood, serum biochemistry, coagulation function and virology tests. The training cohort was divided into two groups, namely, patients with or without significant necroinflammatory activity, based on HAI scores. We eventually selected ALT, GGT, PT and ALB as the most valuable diagnostic indexes and established the H value of a simple scoring system for predicting the absence or presence of significant necroinflammatory activity.

The H value predicted significant necroinflammatory activity in the training group when the AUROC value was at least 0.826 (95% CI 0.786–0.866). With an H value cutoff of 0.560 (the max Youden index point), the sensitivity, specificity and diagnosis accuracy were 83.3, 67.0 and 75.6%, independently. These results show that this model has a high accuracy for evaluating significant liver necroinflammation. In this study, we constructed and validated a model and scoring system to distinguish patients with and without significant necroinflammatory activity. Meanwhile, our aim for this model is to render liver biopsy unnecessary in a considerable proportion of CHB patients. In recent years, many studies have aimed to identify an optimal model for the noninvasive diagnosis of liver fibrosis. However, the noninvasive assessment of liver necroinflammatory activity in CHB patients is less well studied. Therefore, we further improved the model by referring to the research methods adopted by other researchers to diagnose liver fibrosis [12, 21]. We selected low (0.375) and high (0.720) cut-off values according to the Youden index, sensitivity and specificity to identify the absence and presence of significant necroinflammatory activity. At an H index cut-off of 0.375, patients without significant necroinflammatory activity were correctly identified by a high NPV. The H value cut-off of 0.375 was used as an NPV, the sensitivity of diagnosing of liver necroinflammatory activity approached 90.8%. At the same time, patients with significant necroinflammatory activity were diagnosed correctly at an H index cut-off of 0.720 due to the high PPV. The H value cut-off was used as a PPV, the specificity of determining the absence of significant necroinflammatory activity reached 92%. With a combination of low and high cutoff values, the model would provide a more accurate diagnosis. In addition, with more CHB patients, this model would render liver biopsy unnecessary.

The indexes used to establish the model are commonly used clinical tests. ALT is the simplest and most commonly used enzyme for assessing hepatic parenchymal cell injury. In this study, the AUROC of ALT in assessing hepatic necroinflammatory activity in CHB patients was 0.669. Increased serum ALT is an independent risk factor for liver necroinflammatory activity [22]. The serum ALT level in CHB patients should be checked regularly [23]. The use of ALT alone to assess necroinflammatory activity in hepatitis B is not ideal [24], and the ALT level was normal over the long term in 37% of CHB patients with significant liver necroinflammatory activity. Thus, ALT should be combined with other indicators. GGT reflects the degree of liver necroinflammatory activity with a high sensitivity but low specificity. The AUROC value of GGT was 0.771, the highest of all the individual indicators. Elevated GGT in CHB and CHC patients is often associated with bile duct injury [25]. GGT is used in many noninvasive diagnostic models, including the Forns index, the Fibrotest, and the S index. In this study, GGT was found to be an important index with high accuracy in our predictive model. The Child–Pugh classification, which includes ALB and PT, is useful for evaluating liver function reserve and the degree of liver cirrhosis. ALB and PT often decrease with increased liver necroinflammatory activity, indicating that these parameters might reflect the severity of necroinflammatory activity and therefore have greater significance. According to guidelines for the prevention and treatment of chronic hepatitis B published in 2017 by EASL, antiviral treatment should be considered for patients whose liver histology reveals a Knodell HAI ≥ 4 or inflammatory necrosis stage ≥ G2; therefore, the H value is useful for distinguishing between the absence and presence of significant necroinflammatory activity.

Myers et al. assessed 209 CHB patients using the ActiTest model and found that the diagnostic accuracy remained high. The AUROC was 0.82 ± 0.04 [26], but the model, which includes alpha 2-macroglobulin and haptoglobin, can only be used in a few medical institutions and laboratories. The model established in our study is superior to the ActiTest model not only because of the diagnostic accuracy but also due to the lower cost and ease of access. In addition, the parameters in our model exhibit high reproducibility in the clinic. The number of samples included this study is larger than those used in other studies. Similar to other noninvasive diagnostic models, the H value could reduce the requirement of liver biopsy.

Chen et al. assessed 200 CHB patients with cirrhosis using a model constructed by six variables (AST, TBIL, TBA, PT, APRI and serum HBV-DNA) and found that the diagnostic accuracy of this model was high. The AUROC was 0.859 [27], but this model can only be applied to patients with cirrhosis. Thus, whether this model is useful for CHB patients without cirrhosis requires further study. Some advantages of our study include the larger cohort, its applicability to all CHB patients, and the lower cost and ease of access of our model.

However, we should acknowledge that the H value, like other models, has some deficiencies and requires further improvement. First, although it can distinguish somewhat between patients with or without significant necroinflammatory activity, the H value might not be able to accurately grade the necroinflammatory activity or provide prognostic information for CHB patients. Second, even though definite values have been set for the diagnostic model, the diagnostic accuracy is not high enough to predict significant necroinflammatory activity in all cases. As it cannot correctly diagnose liver necroinflammatory activity in all CHB patients, the model would need to be combined with liver biopsy or other diagnostic tests. Finally, more indicators need to be screened and more cases need to be assessed to verify the diagnostic performance of the model.

## Conclusions

The model established in this study is especially useful for evaluating necroinflammatory activity and monitoring therapy outcomes since few patients are willing to have repeated liver biopsies. The H index has excellent diagnostic value for hepatic necroinflammatory activity and appears to be a good noninvasive panel for assessing liver inflammatory activity.

## Abbreviations

HBV: hepatitis B virus
HCV: hepatitis C virus
CHB: chronic hepatitis B
CHC: chronic hepatitis C
HCC: hepatocellular carcinoma
HBsAg: hepatitis B surface antigen
HAI: histologic activity index
APRI: aminotransferase to platelet ratio index
HE: hematoxylin and eosin
RBC: red blood cell
WBC: white blood cell
PLT: platelets
HB: haemoglobin
TG: triglyceride
TC: total cholesterol
ALT: alanine aminotransferase
AST: aspartate aminotransferase
GGT: γ-glutamyl transpeptidase
ALP: alkaline phosphatase
TB: total bilirubin
DB: indirect bilirubin
ALB: albumin
AFP: α-fetal protein
PT: prothrombin time
SEN: sensitivity
SPE: specificity
PPV: positive predictive values
NPV: negative predictive values
LR+: positive likelihood ratios
LR−: negative likelihood ratios
AUROC: the area under the receiver operating characteristic curve
